# Two Distinct Mechanisms Leading to Loss of Virological Control in the Rare Group of Antiretroviral Therapy-Naive, Transiently Aviremic Children Living with HIV

**DOI:** 10.1128/JVI.01535-21

**Published:** 2022-01-26

**Authors:** Vinicius A. Vieira, Emily Adland, Nicholas E. Grayson, Anna Csala, Fa’eeda Richards, Cherrelle Dacon, Rohin Athavale, Ming-Han Tsai, Reena D’Souza, Maximilian Muenchhoff, David Bonsall, Pieter Jooste, Philip J. R. Goulder

**Affiliations:** a Department of Paediatrics, University of Oxford, Oxford, United Kingdom; b Department of Paediatrics, Kimberley Hospital, Kimberley, South Africa; c Antibody Biology Unit, Laboratory of Immunogenetics, National Institute of Allergy and Infectious Diseases, National Institutes of Health, Rockville, Maryland, USA; d Max von Pettenkofer Institute & Gene Center, Virology, National Reference Center for Retroviruses, LMU München, Munich, Germany; e German Center for Infection Research, Munich, Germany; f Big Data Institute, Li Ka Shing Centre for Health Information and Discovery, Nuffield Department of Medicine, University of Oxford, Oxford, United Kingdom; g Wellcome Centre for Human Genetics, University of Oxford, Oxford, United Kingdom; h HIV Pathogenesis Programme, Doris Duke Medical Research Institute, Nelson R Mandela School of Medicine, University of KwaZulu-Natal, Durban, South Africa

**Keywords:** HIV, pediatric, viral control, T-cell response, viral escape

## Abstract

HIV-specific CD8^+^ T cells play a central role in immune control of adult HIV, but their contribution in pediatric infection is less well characterized. Previously, we identified a group of ART-naive children with persistently undetectable plasma viremia, termed “elite controllers,” and a second group who achieved aviremia only transiently. To investigate the mechanisms of failure to maintain aviremia, we characterized in three transient aviremic individuals (TAs), each of whom expressed the disease-protective HLA-B*81:01, longitudinal HIV-specific T-cell activity, and viral sequences. In two TAs, a CD8^+^ T-cell response targeting the immunodominant epitope TPQDLNTML (Gag-TL9) was associated with viral control, followed by viral rebound and the emergence of escape variants with lower replicative capacity. Both TAs mounted variant-specific responses, but only at low functional avidity, resulting in immunological progression. In contrast, in TA-3, intermittent viremic episodes followed aviremia without virus escape or a diminished CD4^+^ T-cell count. High quality and magnitude of the CD8^+^ T-cell response were associated with aviremia. We therefore identify two distinct mechanisms of loss of viral control. In one scenario, CD8^+^ T-cell responses initially cornered low-replicative-capacity escape variants, but with insufficient avidity to prevent viremia and disease progression. In the other, loss of viral control was associated with neither virus escape nor progression but with a decrease in the quality of the CD8^+^ T-cell response, followed by recovery of viral control in association with improved antiviral response. These data suggest the potential for a consistently strong and polyfunctional antiviral response to achieve long-term viral control without escape.

**IMPORTANCE** Very early initiation of antiretroviral therapy (ART) in pediatric HIV infection offers a unique opportunity to limit the size and diversity of the viral reservoir. However, only rarely is ART alone sufficient to achieve remission. Additional interventions that likely include contributions from host immunity are therefore required. The HIV-specific T-cell response plays a central role in immune control of adult HIV, often mediated through protective alleles such as HLA-B*57/58:01/81:01. However, due to the tolerogenic and type 2 biased immune response in early life, HLA-I-mediated immune suppression of viremia is seldom observed in children. We assessed a rare group of HLA-B*81:01-positive, ART-naive children who achieved aviremia, albeit only transiently, and investigated the role of the CD8^+^ T-cell response in the establishment and loss of viral control. We identified a mechanism by which the HIV-specific response can achieve viremic control without viral escape that can be explored in strategies to achieve remission.

## INTRODUCTION

Intrauterine transmission of HIV usually occurs at the end of pregnancy (1), allowing antiretroviral therapy (ART) initiation at birth, earlier in the course of infection than following adult horizontal transmission. Early ART rapidly stops viral seeding and limits the size and diversity of the viral reservoir (2, 3). Thus, infants with early suppression may be better placed to become posttreatment controllers after a period of time on ART than adults (4). However, ART alone is not sufficient to achieve remission, other than in rare well-documented exceptions to the rule (5–7), and additional interventions are therefore necessary before ART is stopped (8, 9). Ultimately, host innate and/or adaptive immunity will likely need to contribute to maintain aviremia following treatment interruption.

In adults, HIV-specific CD8^+^ T-cell responses play a central part in immune control of HIV (10–13), with important contributions from other arms of the immune system, including NK cells (14–17). In children living with HIV, although HIV-specific cytotoxic T-lymphocyte (CTL) responses are detectable from birth (18, 19), due to the non-Th1-polarizing nature of early life immunity (20, 21), these are of lower breadth and do not impose strong selection pressure on the virus until midchildhood (22). In pediatric HIV infections, a viral setpoint is observed only after the age of 5 years in those who survive without ART (23). Although as many as 10% of ART-naive children living with HIV are pediatric slow progressors (PSPs) (24–26), having maintained normal-for-age CD4^+^ T-cell counts at 5 years, pediatric elite control (EC; undetectable plasma viremia in ART-naive individuals) is extremely rare (27). Pediatric individuals exhibiting EC are at least 10-fold less common than adults, with a strong bias toward the female sex, and achieve suppression of viremia only after 5 to 10 years of infection (27). PSPs are usually viremic, but those with better viral control are marked by a CD8^+^ T-cell response directed to the conserved Gag/Pol regions rather than to Env, Nef, or the other accessory proteins (Tat, Rev, Vif, Vpr, and Vpu) (28).

The effect of HLA-B molecules in immune control of HIV is substantially stronger in adult than in pediatric infection (22). However, even in adults, HLA-I polymorphisms explain only 20% of differences in plasma viral load (29). Although 40 to 50% of individuals exhibiting EC express HLA-B*57 (30), since EC prevalence is approximately 0.3% (31) and HLA-B*57 prevalence in most populations is approximately 7%, <5% of adults living with HIV and expressing HLA-B*57 exhibit EC. Other factors influencing CD8^+^ T-cell function clearly also play a role in immune control, however. Notably, HIV-specific CD8^+^ T-cells in those with EC have higher proliferative capacity and are more polyfunctional than in noncontrollers targeting the same epitopes (32–36).

We previously described the rare group of ART-naive children who met the criteria for EC, namely, a minimum of three consecutive tests showing undetectable plasma HIV-RNA copies spanning at least a year in the absence of ART (31, 37). We also identified an uncommon group of ART-naive children who had transient episodes of aviremia but who did not maintain viral suppression sufficiently long-term to meet the criteria for EC (27). To further assess the mechanisms linked with failure to maintain aviremia in these children, we characterized the HIV-specific T-cell responses and carried out longitudinal deep sequencing of the virus in three transiently aviremic children (TAs), each of whom expressed HLA-B*81:01, an allele strongly associated with immune control HIV in the adult African populations (29, 38, 39).

## RESULTS

We previously identified a total of 21 TAs in different cohorts around the globe comprising >12,000 children living with HIV (27). TAs were defined as those with spontaneous plasma HIV RNA viral load below the limit of detection (<50 copies/mL) on at least one occasion, but without fulfilling the criteria for EC. In the cohort of 21 TAs, there was a preponderance of females (62%), as previously described for PSPs (40). The median age at undetectable viremia varied from 1.8 to 15.1 years (median, 7.4 years; interquartile range [IQR 4.6 to 12.4]). Undetectable viremia was detected in a single time point for all except for four individuals. Here, we describe in detail three TAs expressing the disease-protective HLA-B*81:01.

**FIG 1:**
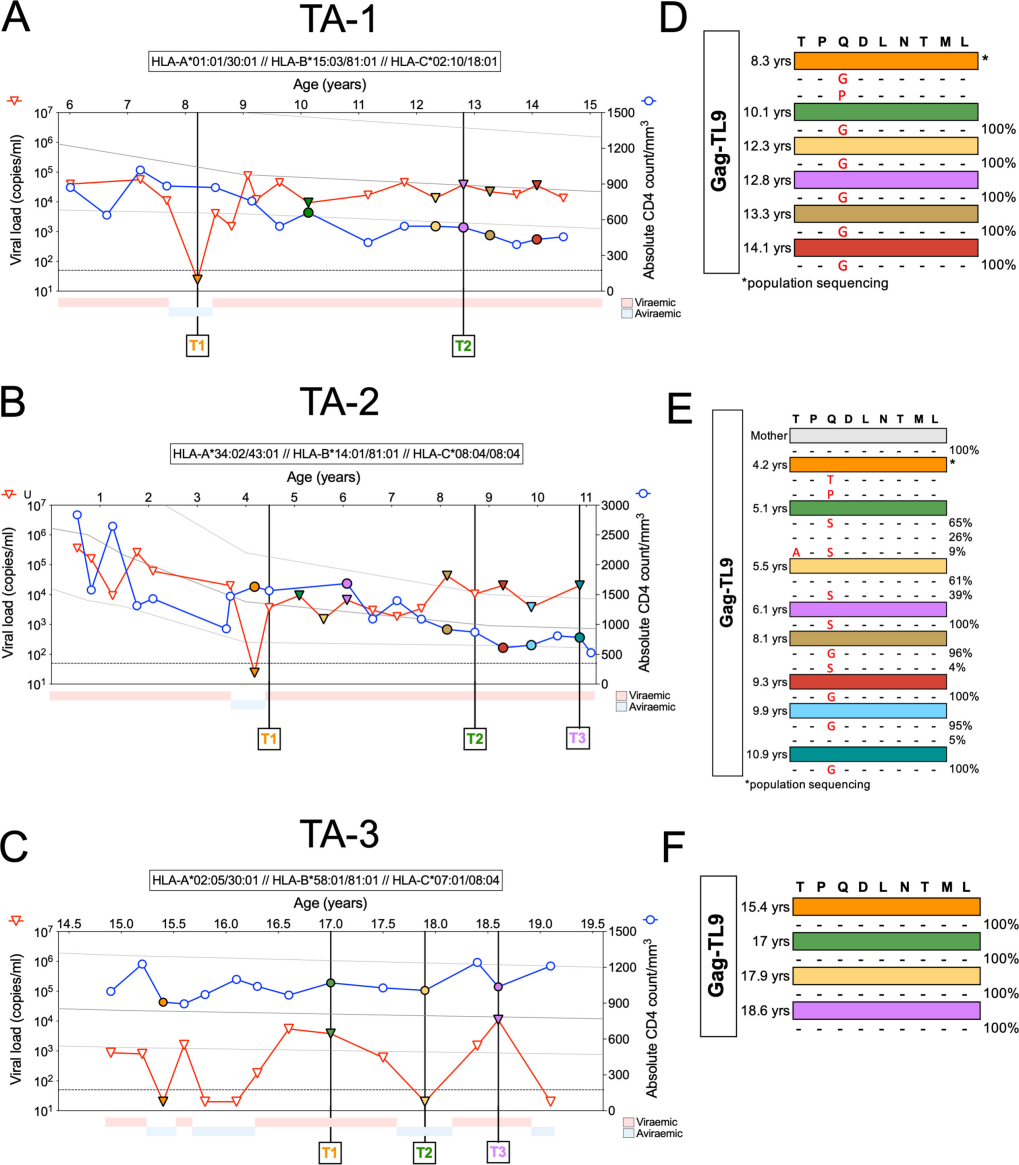
Longitudinal clinical data and escape in the dominant TL9-Gag epitopes after transient aviremia. Plasma HIV-RNA load (red triangles) and absolute CD4^+^ T-cell count (blue circles) are shown for TA-1 (A), TA-2 (B), and TA-3 (C). The horizontal dashed line for plasma HIV RNA copies represents the limit of 50 copies/mL. The 10th, 50th, and 90th percentiles for absolute CD4^+^ T-cell count for HIV-uninfected children are represented by the three gray lines. (D to F) Population (28) and deep sequencing for the TL9-Gag epitope. Colors match the corresponding symbols in panels A to C. Time point 1 (T1) to T3 are the time points selected for the avidity assay in Fig. 4.

TA-1 and TA-2 were both female ART-naive children from Kimberley, South Africa, in whom we had previously reported selection of escape mutants within the HLA-B*81:01-restricted Gag epitope TPQDLNTML (Gag 180 to 188; TL9), followed by the emergence of TL9 variant-specific responses (28). In both these cases, we observed transient aviremia followed by viral rebound and CD4^+^ T-cell decline. TA-1 reached aviremia at the age of 8.2 years, rebounding within 3 months to a plasma viral load of 4,100 copies/mL (Fig. 1A). Within 9 months, her viral load had returned to the preexisting set point (median of 17,294 copies/mL), which was now followed by a progressive decline in her CD4^+^ T-cell count. TA-2 was followed from birth (Fig. 1B). Similar to TA-1, but at a younger age, TA-2 reached undetectable viremia at the age of 4.2 years, within 3 months rebounding to 3,100 copies/mL. Within 9 months, a new viral set point was maintained at 9,100 copies/mL, and as in TA-1, this was followed by a decline in the CD4^+^ T-cell count. TA-3, also HLA-B*81:01-positive, and an older male from Durban, South Africa, followed from 14.8 years, reached undetectable viremia four times across 4.5 years of follow-up, but without achieving the criteria for EC (Fig. 1C). His median plasma viremia was 700 copies/mL, and he maintains a healthy CD4^+^ T-cell count to date, most recently 1,200 CD4^+^ T cells/mm^3^. Whereas TA-1 and TA-2 have both now started ART, TA-3 has, despite medical advice, opted to remain ART naive.

**FIG 2:**
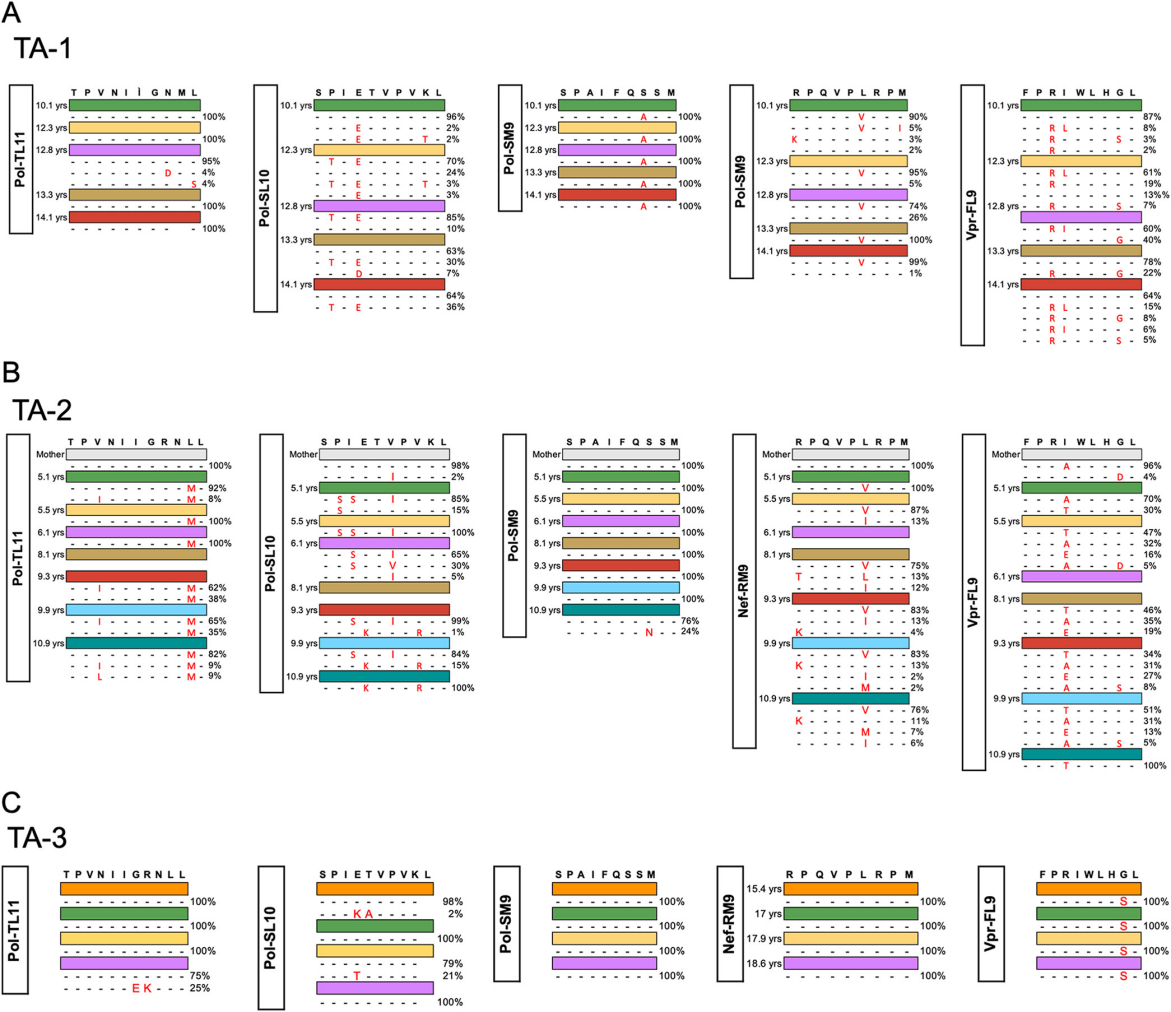
Deep sequencing for HLA-B*81:01-targeted epitopes. Deep sequencing data are shown for well-known HLA-B*81:01-targeted epitopes for TA-1 (A), TA-2 (B), and TA-3 (C). Time point colors match those in Fig. 1.

We had previously reported the results of population sequencing of the virus encoding the immunodominant HLA-B*81:01-restricted TL9 epitope from two time points available in TA-1 and TA-2 (28). To achieve better resolution of the kinetics of viral selection pressure in parallel with HIV-specific CD8^+^ T-cell response, here we carried out deep sequencing of isolates from a further five and seven time points, respectively, for TA-1 and TA-2 and 4 time points for TA-3. In TA-1, following the initial time point studied at 8.3 years, the viral sequence was 100% dominated by the Q182G variant (Fig. 1D). In TA-2, where follow-up extended over more than 11 years from infancy, the Q182G variant also reached 100% fixation, but in this case via Q182T and subsequently Q182S (Fig. 1E). In contrast, deep sequencing of the virus from TA-3 demonstrated no evidence of any selection of escape mutants in Gag-TL9, despite the multiple viral rebounds (Fig. 1F). Both TA-1 and TA-2 also exhibited selection of escape mutants within the HLA-B*81:01-restricted Pol epitope SPIETVPVKL (Pol 158 to 167; SL10) and the Vpr epitope FPRIWLHGL (Vpr 34 to 42; FL9). Escape mutants also arose in the Pol epitopes SPAIFQSSM (Pol 311 to 319; SM9) for TA-1 and TPVNIIGRNLL (Pol 136 to 146; TL11) for TA-2 (Fig. 2A and B). Similarly, we did not find any evidence in TA-3 of escape mutations in any of the known HLA*81:01 restricted epitopes, including Pol-SL9, Pol-TL11, Nef-RM9, and Vpr-FL9, except for the G41S variant in the Vpr FL9 epitope (Fig. 2C).

**FIG 3:**
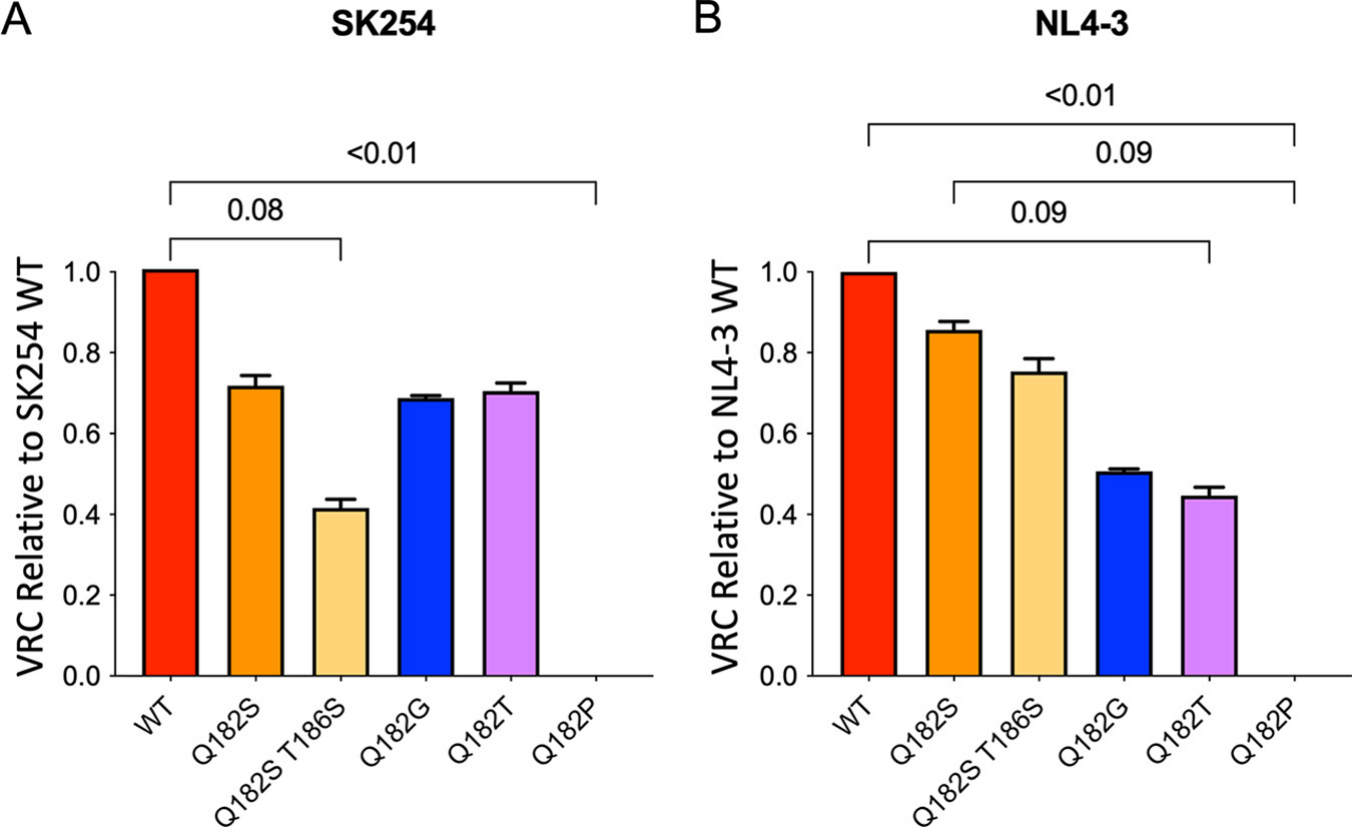
Lower viral replication in TL9 escape variants. Viruses containing a single Q182X mutation or Q182S/T186S were constructed, and VRC was measured compared to the growth of a WT virus in C clade (A) and B clade (B) infections. Triplicates were compared using a Friedman test to obtain the exact *P* value.

To investigate why these particular TL9 escape mutants arose in TA-1 and TA-2 and, in the case of Q182G, were maintained, even though these were the best-recognized variants, we evaluated the impact of the TL9 escape variants on virus replication, comparing variant viral growth against either NL4-3 or a chimeric consensus C clade Gag-Pro/NL4-3 virus (Fig. 3A and B). The Q182P variant was detected as a low-frequency variant at the aviremic time point in both TA-1 and TA-2 via population proviral DNA sequencing, but because plasma viral RNA at these aviremic time points could not be amplified, no RNA sequence was available. The Q182P variant reduced viral replication capacity to beneath detectable limits, explaining its temporary appearance only. The more common TL9 escape variants decreased viral replicative capacity (VRC) but less dramatically, without crippling the virus, in both B and C clade Gag backbones, but the VRC reductions were broadly similar for all of these more common variants. Hence, the selection of Q182G rather than Q182T or Q182S is likely the result of the relative benefit of the escape mutant in evading the CTL response.

**FIG 4:**
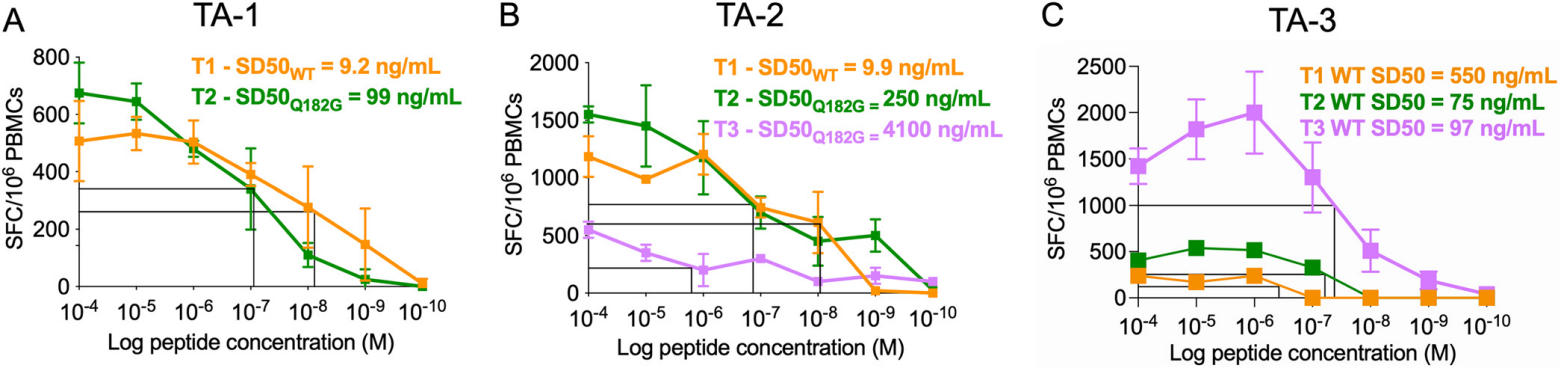
Lower avidity in the T182G-specific CD8^+^ T-cell response. Avidity curves of the dominant TL9 CD8^+^ T-cell response determined by IFN-γ ELISPOT are shown for the WT and selected escape T182G mutants for TA-1 (A) and TA-2 (B) at different time points. Avidity curves of the WT TL9 epitope in different time points are shown for TA-3 (C).

To investigate this further, we compared the functional avidity of CTL responses to the wild type (WT) TL9 at early time points with that of the Q182G-specific response at later time points. In both TA-1 and TA-2, the functional avidity of the Q182G-specific response was substantially lower, with an SD50 (the concentration of peptide needed to achieve half maximal response) 1.03 and 2.62 log_10_ higher than that of the WT during viremic control (Fig. 4A and B). This lower functional avidity of the Q182G-specific CTL compared with WT TL9-specific CTL is therefore consistent with the failure to control viremia and to prevent CD4^+^ T-cell decline in these two transiently aviremic children, despite initially cornering the virus via the variant-specific CTL response (28). These findings are also consistent with studies of HLA-B*81:01-positive adults showing emergence of both Q182S and Q182G variants in association with HIV disease progression (41).

**FIG 5:**
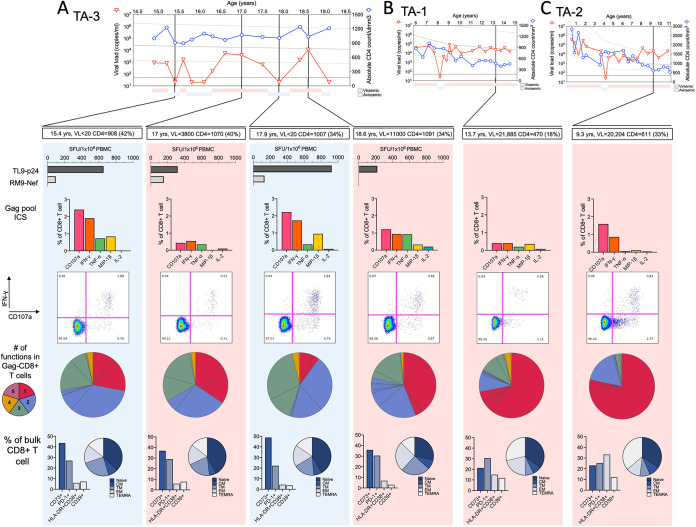
Fluctuations in the magnitude and quality of CD8^+^ T-cell response determine viral control during transient aviremia in TA-3. Longitudinal data showing plasma HIV-RNA load (red triangles) and absolute CD4^+^ T-cell count (blue circles) for TA-3 (A), TA-1 (B), and TA-2 (C). Magnitudes of TL9 CD8^+^ T-cell responses determined by IFN-γ ELISPOT are shown for TA-1, followed by intracellular cytokine staining of CD107a, IFN-γ, TNF-α, MIP-1β, and IL-2; polyfunctionality showing coexpression of markers on CD8^+^ T cells after Gag-stimulated PBMCs; memory subset distribution; and expression of activation/exhaustion markers (CD73, PD-1, CD38, HLA-DR, and CD39).

To identify the mechanism of viral rebound in TA-3, we investigated whether functional differences in HIV-specific CD8^+^ T-cell responses were present at aviremic time points compared with subsequent time points when viral rebound had occurred. Viral rebound was associated with a >3-fold decrease in the magnitude of the TL9-specific interferon gamma (IFN-γ) secreting CD8^+^ T-cell enzyme-linked immunospot assay (ELISPOT) response and a >2-fold decrease in the intracellular cytokine staining (ICS) response magnitude (Fig. 5A). Functional avidity of the TL9 response was also somewhat decreased at viremic time points (Fig. 4C). Degranulation (CD107a), IFN-γ and MIP-1β expression were all decreased during the periods of viral rebound, and responses were less polyfunctional (defined as 2 or more functions) during the viremic time points. As previously observed in pediatric nonprogressors (23), despite changes in plasma viral load, levels of immune activation (HLA-DR^+^ CD38^+^) were unaltered, and exhaustion (PD-1^+^ and CD39^+^) markers likewise were unaffected. High levels of CD73, a marker associated with memory preservation (42, 43), were also unchanged by fluctuations in viremia. When the viremic period was examined, TA-1 and TA-2 also showed a lower proportion of Gag-specific CD8^+^ T-cells performing more than 2 functions, a higher frequency of terminal effector (TEMRA) cells, and higher levels of CD38^+^ HLA-DR^+^ expression on CD8^+^ T cells (Fig. 5B and C) than TA-3.

In contrast to TA-1 and TA-2, in whom attainment of aviremia had occurred only once and was followed by CTL escape, persistent viremia and CD4^+^ T-cell decline, TA-3 transitioned from total and partial control of viremia in association with fluctuations in the quality of the CD8^+^ T-cell response, no selection of CTL escape, and maintenance of highly stable and normal-for-age absolute CD4^+^ T-cell counts.

## DISCUSSION

In this report, we describe three ART-naive pediatric slow progressors who all express the disease-protective HLA-B*81:01 and who controlled viremia to undetectable levels but only transiently. We identified two distinct mechanisms linked to loss of viral control: (i) the selection of an escape variant that evaded the CD8^+^ T-cell response, leading to viral rebound (TA-1 and TA-2), and (ii) fluctuations in the magnitude and quality of the CD8^+^ T-cell response associated with differential levels of viral control (TA-3).

Through childhood, the HIV-specific CD8^+^ T-cell response becomes increasingly more effective at killing infected targets and progressively strengthens pressure for the selection of CTL escape mutants (28). Previously, we demonstrated that PSPs with immune control of HIV had lower magnitude and breadth of HIV-specific CD8^+^ T-cell responses than viremic noncontrollers but effectively targeted conserved epitopes in Gag and Pol, adopting a cornering strategy by mounting a new response to the selected escaped variants (28). This strategy drives the virus down an evolutionary cul-de-sac with progressive loss of viral fitness, which it constantly needs to escape further to avoid variant-specific CTL responses. TA-1 and TA-2 are clear examples of the cornering strategy seen in children who mounted a response against the selected variants of Gag-TL9 epitope and induced the fixation of the Q182S and Q182G mutants with lower replicative capacity. However, in these children, the new responses failed to control viremia completely and led to a progressive decline in the CD4^+^ T-cell count. In neither of these two cases was the more frequently observed escape mutation at Gag-186 T186S or the less common mutation T186M (44–46) selected; these reduce VRC substantially more than the common variants at Gag-182 (28, 47). This may account for their short period of viremic control in these two children and subsequent CD4^+^ T-cell decline. In these two cases, the escape mutant selected did not damage viral replication excessively and the variant-specific responses had lower functional avidity than the WT-specific responses. In the HLA-B*81:01-positive child described previously (28), for whom cornering of the virus was described in association with maintained normal-for-age CD4^+^ T-cell counts, T186M had been selected, crippling viral replication, and at the same time the variant-specific response had the same functional avidity as the WT-specific response.

The mechanism identified in TA-3 seems to be more sustained in the long-term. In this case, the child was enrolled at the age of 14 years and had reached adolescence with a healthy CD4^+^ T-cell count and a median plasma viral load below 1,000 copies/mL. The periods of detectable viremia were marked by a decrease in both the magnitude and quality of the specific CD8^+^ T-cell response rather than by the selection of an escape variant. The mechanism seen here is reminiscent of that described in pediatric individuals with EC, where viral control is mediated by a highly effective polyfunctional Gag-specific CD8^+^ T-cell response while low immune activation and exhaustion levels are maintained (48). This finding is consistent with previous work in adults showing improved viral control in association with superior qualitative aspects of the immune response (32, 34–36, 49). Indeed, most adults living with HIV expressing the protective HLA-B*57 allele progress to AIDS despite having access to the same HLA-B*57-restricted epitopes as HLA-B*57-positive individuals with EC (30). The polyfunctionality of the HIV-specific response is a common feature of adults with EC, irrespective of the HLA class I molecules expressed (33).

The mechanism behind the fluctuations in the Gag-specific CTL effector function is unknown. We can speculate that the polyfunctionality and efficacy of the CTL response in such a pediatric nonprogressor are affected by rapid changes in regulatory mechanisms, mainly via expression of immune checkpoint inhibitors, that are in turn the consequence of intercurrent illnesses or other immune stresses. As a consequence of the decreased CTL functionality, the viral load can climb. Once the immune stress has passed and CTL polyfunctionality across a broad range of epitopes is allowed to resume, immune control is once again achieved without escape. Some evidence for this is the fluctuation in the expression of CD73 and PD-1 on CD8^+^ T cells. The more usual scenario is one in which CTL functionality is diminished by declining CD4^+^ T-cell counts and function, opening the door to viral escape as effective suppression of viral replication gradually eases off. Viral escape does not occur in the scenario exemplified by TA-3 because there is no effective response to escape from if the regulatory mechanism operates sufficiently quickly.

It is important to note the limitations of the current study. First, the cases described are necessarily anecdotal, given the rarity of elite control and even transient aviremia in pediatric infection (27). Second, because of sample availability, we had to focus on the immunodominant HLA-B*81:01-restricted TL9 response rather than evaluating other virus-specific responses also. Despite this, the deep sequencing approach allowed us to observe differences between TA-1/TA-2 and TA-3 in terms of escape mutants across all the HLA-B*81:01-restricted CTL epitopes. We could not assess in this study the direct ability of variant-specific CTLs to inhibit replication of the variant virus compared to the ability of the WT-specific CTLs to inhibit replication of the WT virus. Although this was a definitive experiment, we were limited by sample availability and the necessity of having variant-specific and WT clones to achieve this. Finally, the altered magnitude and quality of the CTL responses in TA-3 in association with fluctuations in viremia could be the cause or consequence of viremic changes. Nonetheless, the immunogenetic data showing that HLA-B*81:01 protects against HIV disease progression support the hypothesis that the HLA-B*81:01-restricted TL9 responses strongly influence immune control of HIV (50).

Even if anecdotal, the cases described add new insights into the mechanisms of viremic control in children (22, 23, 28). The cornering approach is more commonly observed in children than in adults (28) and was modeled previously as a vaccine strategy (51) by leading the virus down an evolutionary cul-de-sac to a variant with low replicative capacity that is still well recognized by a variant-specific CTL response. However, if the variant is sufficiently fit and the variant-specific response is less efficacious, disease progression will result. A robust HIV-specific T-cell response of high quality that is capable of efficiently controlling viral replication would appear to be more sustainable in the long term. Although in TA-3 the common escape variants are less evident than the WT, it may be that when the CTL response is most efficacious, replication is suppressed and variants are not generated, and when the CTL response is less potent, the small selective advantage of the escape mutant is outweighed by the high cost to viral replicative capacity. These findings support the development of therapeutic models that prioritize induction of high-quality (i.e., robust and polyfunctional) HIV-specific T-cell responses that target immunodominant and conserved epitopes.

## MATERIALS AND METHODS

### Clinical and demographic data.

All participants or their caregivers provided informed consent at enrollment. The study was approved by Free State Ethics Committee, Bloemfontein, South Africa; the Biomedical Research Ethics Committee, University of KwaZulu-Natal, Durban, South Africa; and the Oxford Research Ethics Committee. The CD4^+^ T-cell count and plasma HIV-RNA viral loads were measured as part of their clinical follow-up using flow cytometry and the Roche Amplicor version 1.5 assay, respectively.

### Host genetics: HLA typing.

Samples from study subjects were HLA-A, -B, and ‐C sequence base typed in the Clinical Laboratory Improvement Amendments (CLIA)/American Society for Histocompatibility and Immunogenetics (ASHI)-accredited laboratory of William Hildebrand (Association of British HealthTech Industries [ABHI]) at the University of Oklahoma Health Sciences Center using a locus-specific PCR amplification strategy and a heterozygous DNA sequencing method for exons 2 and 3 of the class I PCR amplicon. Relevant ambiguities were resolved by homozygous sequencing. DNA sequence analysis and HLA allele assignment were performed with the software Assign-SBT v3.5.1 from Conexio Genomics.

### Cellular immunology. (i) T-cell immunophenotype.

Thawed peripheral blood mononuclear cells (PBMCs) were rested in R10 overnight at 37°C in 5% CO_2_. One million cells were used to characterize the T-cell compartment by assessing memory, activation, and exhaustion markers. After staining at room temperature for 30 min with Live/Dead near-infrared (IR) stain (Invitrogen) according to the manufacturer’s instructions, cells were incubated for another 30 min at 4°C in fluorescence-activated cell sorting (FACS) buffer containing the antibodies against CD3 (BV605; UCHT1; BioLegend), CD4 (BV650; RPA-T4; BioLegend), CD8a (BV570; RPA-T8; BioLegend), CD45RA (Alexa Fluor 700; H100; BioLegend), CCR7 (Pacific Blue; G043H7; BioLegend), CD27 (BV510; M-T271; BioLegend), CCR5 (PE-Cy7; HM-CCR5; BioLegend), HLA-DR (APC-R700; G46-6; BD), CD38 (PerCP/Cy5.5; HIT2; BioLegend), CD39 (APC; A1; eBioscience), CD73 (PE; AD-2; BioLegend), and PD-1 (PE-eFluor610; J105; eBioscience). Cells were washed and fixed in 2% paraformaldehyde (PFA) and acquired on a BD LSR II. The data were analyzed in FlowJo v10.6.2 (TreeStar LLC).

### (ii) HIV specific response.

One million PBMCs were stimulated with a Gag pool of overlapping clade C consensus peptides (NIH AIDS Reagent Program) at a final concentration of 2 μg/mL in the presence of anti-CD28/anti-CD49d (BD) and anti-CD107a (PE-Cy7; H4A3; BioLegend) and incubated for 1 h when brefeldin A and monensin (BioLegend) were added, followed by another 5-h incubation. Surface staining was performed as described above with Live/Dead, CD3, CD4, and CD8. Cells were permeabilized with Cytofix/Cytoperm solution (BD) and stained with antibodies against IFN-γ (PE-Dazzle594; B27; BioLegend), interleukin 2 (IL-2) (BV510; MQ1-17H12; BioLegend), tumor necrosis factor alpha (TNF-α) (Alexa Fluor 700; MAb11; BioLegend), and MIP-1β (PE; D21-1351; BD) for 30 min at 4°C. Cells were washed and acquired on a BD LSR II instrument. The data were analyzed in FlowJo v10.6.2 (TreeStar LLC).

IFN-γ ELISPOTs were performed as previously described using overlapping peptides (18-mers with a 10-amino-acid overlap) based on the clade C consensus sequence in conjunction with optimal peptides defined by the corresponding HLA-I type (19, 52). Briefly, 96-well plates with a polyvinylidene fluoride membrane (Millipore) were coated with anti-human monoclonal antibody (Mabtech) in phosphate-buffered saline (PBS) and incubated at 4°C overnight. Before starting the assay, the plate was washed with blocking buffer (PBS and 1% fetal calf serum [FCS]). Fifty thousand or 100,000 PBMCs and the peptides at a final concentration of 2 μg/mL were added to the wells. Negative and positive controls included dimethyl sulfoxide (DMSO) at the same concentration as the peptide pools and phytohemagglutinin (PHA; Sigma). After 14 to 16 h incubation at 37°C in 5% CO_2_, IFN-γ-producing cells were revealed after subsequent washes with PBS and incubations with biotinylated anti-IFN-γ antibody (Mabtech), streptavidin-alkaline phosphatase conjugate antibody (Mabtech), and alkaline phosphatase substrate reagents (Bio-Rad). The last reaction was stopped by washing the plates with tap water and leaving them to air dry overnight. The number of spots per well was quantified using an automated ELISPOT plate reader (AID ELISPOT reader system; Autoimmun Diagnostika GmbH, Germany) and manually checked. A response was defined as positive if there were ≥50 spot-forming cells (SFC)/million PBMCs after background subtraction (mean of the negative controls plus 3 standard deviations).

To determine the recognition and magnitude of wild-type (WT) TL9 epitope and autologous variants, assays were performed in triplicate at peptide concentrations of 10^−10^ M to 10^−4^ M. Data shown are means of the response after background subtraction.

### Virology. (i) Viral sequencing.

Sanger sequencing were generated from genomic DNA extracted from whole blood and amplified by nested PCR. Sequencing was undertaken using the BigDye ready reaction terminator mix (V3) (Department of Zoology, University of Oxford). Sequence data were analyzed using Sequencher v.4.8 (Gene Codes Corporation). Nucleotides for each gene were aligned manually in Se-Al v.2.0a11.

Next-generation sequencing (NGS) was performed as previously described (53). Briefly, plasma samples were thawed at room temperature and total RNA was extracted using the NucliSens easyMAG system (bioMérieux) and concentrated with Agencourt RNAClean XP (Beckman Coulter). Libraries were prepared using the SMARTer stranded total RNA-Seq kit, v2 (Pico Input Mammalian; TaKaRa Bio). The RNA was denatured at 72°C with tagged random hexamers, followed by cDNA synthesis using in-house sets of indexed primers (54). Ribosomal cDNA was not depleted. Five microliters of each amplified library was pooled, and shorter libraries were eliminated with a lower ratio than recommended of Agencourt AMPure XP. A high-sensitivity D1000 ScreenTape assay on a TapeStation system (Agilent) and a Qubit double-stranded DNA (dsDNA) HS assay (Thermo Fisher Scientific) were used to measure size distribution and concentration. A total of 500 ng of pooled libraries was hybridized (SeqCap EZ reagent kit; Roche) to a mixture of custom HIV-specific biotinylated 120-mer oligonucleotides (xGen Lockdown Probes, Integrated DNA Technologies, Coralville, IA, USA) and then captured by streptavidin-conjugated beads. Unbound DNA was washed off (SeqCap EZ hybridization and wash kit; Roche), and the remaining libraries were then PCR amplified for sequencing using a MiSeq instrument (Illumina), resulting in read lengths up to 300 nucleotides (paired end).

To obtain the epitope ratios from NGS reads, initially the software Trimmomatic (55) was used to trim Illumina adaptors and low-quality data from the raw reads. Where possible, reads were then merged using a minimum overlap of 10 nucleotides. The processed reads were then subjected to BLAST searching against the Los Alamos Sequence Database (http://www.hiv.lanl.gov/) HIV-1 “Web Alignments 2017” to obtain the HXB2 alignment positions for each read. Reads that entirely contained the epitope were used to find the epitope ratios at each position.

### (ii) Viral mutagenesis.

Single mutations were introduced into the Gag region of HIV-1 using the B clade NL4-3 plasmid and the patient-derived C clade Gag protease sequence, SK-254 (M), which had been modified to consensus C as previously described (28). Glutamine was replaced with proline, glycine, or threonine at the 182nd position in clade B and 179th position in clade C. This was done using a QuikChange Lightning site-directed mutagenesis kit (Agilent Technologies) using custom primers from Eurofins Genomics. The forward primers are NL4-3 Q182T (5′-GCA TTA TCA GAA GGA GCC ACC CCA ACG GAT TTA AAT ACC ATG CTA AAC ACA-3′), Q182G (5′-GCA TTA TCA GAA GGA GCC ACC CCA GGA GAT TTA AAT ACC ATG-3′), Q182P (5′-AA GGA GCC ACC CCA CCA GAT TTA AAT ACC ATG C-3′), SK-254(M) Q179T (5′-GCA TTA TCA GAA GGA GCC ACC CCA ACG GAT TTA AAC ACC ATG TTA AAT ACA G-3′), Q179G (5′-GCA TTA TCA GAA GGA GCC ACC CCA GGA GAT TTA AAC ACC ATG-3′), and Q179P (5′-GAA GGA GCC ACC CCA CCA GAT TTA AAC ACC ATG T-3′).

All plasmids were prepared using a Maxiprep kit following the manufacturer’s instructions (HiSpeed plasmid maxikit; Qiagen, Hilden, Germany).

### (iii) Viral replicative assays.

A mutated version of pNL4-3 was constructed that lacks the entire Gag and Protease region (Stratagene Quick-Change XL kit), replacing this region with a BstE II (New England Biolabs) restriction site at the 5′ end of Gag and the 3′ end of protease. To generate recombinant viruses, 10 μg of BstEII-linearized plasmid plus 50 μl of the second-round amplicon (approximately 2.5 μg) were mixed with 2 × 10^6^ cells of a Tat-driven green fluorescent protein (GFP) reporter T-cell line (GXR 25 cells) in 800 μl of R10 medium (RPMI 1640 medium containing 10% fetal calf serum, 2 mM l-glutamine, 100 U/mL penicillin, and 100 μg/mL streptomycin) and transfected by electroporation using a Bio-Rad Gene Pulser II instrument (300 V and 500 μF). Following transfection, cells were rested for 45 min at room temperature, transferred to T25 culture flasks in 5 mL warm R10, and fed with 5 mL R10 on day 4. GFP expression was monitored by flow cytometry (FACSCalibur; BD Biosciences), and once GFP expression reached >30% among viable cells, supernatants containing the recombinant viruses were harvested and aliquots stored at −80°C.

The replication capacity of each chimera is determined by infection of GXR cells at a low multiplicity of infection (MOI) of 0.003. The mean slope of exponential growth from day 2 to day 7 was calculated using the semi log method in Excel. This was divided by the slope of growth of the wild-type NL4-3 control included in each assay to generate a normalized measure of replication capacity. Replication assays were performed in triplicate, and results were averaged. These VRC determinations were undertaken by workers entirely blind to the identity of the study subject.
